# Differences regarding the five-factor personality model in patients with subjective cognitive decline and mild cognitive impairment

**DOI:** 10.1007/s40211-018-0292-z

**Published:** 2018-10-17

**Authors:** Evelyn Berger-Sieczkowski, Bernadette Gruber, Elisabeth Stögmann, Johann Lehrner

**Affiliations:** Department of Neurology, Medical University of Vienna, Währinger Gürtel 18–20, 1090 Vienna, Austria

**Keywords:** Subjective cognitive decline (SCD), Mild cognitive impairment (MCI), Non-amnestic MCI (naMCI), Amnestic MCI (aMCI), Alzheimer’s disease (AD), Dementia, Personality, Subjektive kognitive Beeinträchtigung (SCD), Leichte kognitive Beeinträchtigung (MCI), Nicht amnestische MCI (naMCI), Amnestische MCI (aMCI), Alzheimer-Krankheit (AD), Demenz, Persönlichkeit

## Abstract

Personality and dementia are connected in different ways. A broad knowledge about personality and prodromal stages of dementia might be helpful to identify dementia as early as possible. Hence, personality differences between three cognitively impaired groups on the basis of patients’ self-assessments of personality traits and connections between personality and cognitive functioning were examined via a cross-sectional study. The sample consisted of cognitively impaired patients (*N* = 133), aged 50 and older, who came to a memory clinic due to cognitive complaints. The test procedure encompassed a cognitive screening, the Neuropsychological Test Battery Vienna (NTBV), and self-assessment questionnaires such as the Big Five Plus One Persönlichkeitsinventar (B5PO). While patients with subjective cognitive decline (SCD) did not differ from those with non-amnestic mild cognitive impairment (naMCI) concerning the different personality traits, patients with amnestic mild cognitive impairment (aMCI) showed significantly lower scores for extraversion (*p* < 0.05), openness (*p* < 0.001), and empathy (*p* < 0.001) than patients with SCD as well as patients with naMCI. Thus, cognitively impaired groups mainly differ concerning personality traits depending on whether they do show memory decline or not.

## Zusammenfassung

Persönlichkeitsveränderung und Demenz sind miteinander assoziiert. Wissen über Persönlichkeitsveränderungen bei prodromalen Stadien der Demenz könnte hilfreich sein, um Demenz früher zu erkennen. Anhand einer Querschnittsstudie wurden Persönlichkeitsunterschiede zwischen drei kognitiv beeinträchtigten Gruppen anhand der Selbsteinschätzung von Persönlichkeitsmerkmalen untersucht. Des Weiteren wurden Zusammenhänge zwischen Persönlichkeit und kognitiver Funktion untersucht. Die Stichprobe bestand aus kognitiv beeinträchtigten Patienten (*N* = 133) im Alter von 50 Jahren und älter, die aufgrund kognitiver Beschwerden eine Gedächtnisklinik aufsuchten. Die Untersuchung umfasste ein kognitives Screening, die Neuropsychologische Testbatterie Wien (NTBV) und das Big Five Plus One Persönlichkeitsinventar (B5PO). Während sich Patienten mit subjektivem kognitivem Abbau (SCD) nicht von denen mit nicht-amnestischer leichter kognitiver Beeinträchtigung (naMCI) bezüglich der verschiedenen Persönlichkeitsmerkmale unterschieden, zeigten Patienten mit amnestischer leichter kognitiver Beeinträchtigung (aMCI) signifikant niedrigere Werte für Extraversion (*p* < 0,05), Offenheit (*p* < 0,001) und Empathie (*p* < 0,001) als Patienten mit SCD sowie Patienten mit naMCI. Kognitiv beeinträchtigte Gruppen unterschieden sich hinsichtlich Persönlichkeitseigenschaften in Abhängigkeit davon, ob Gedächtnisbeeinträchtigungen vorlagen oder nicht.

## Introduction

For a long time, personality traits have been seen as quite stable from the age of about 30 onwards [1], while later investigations could demonstrate an instability concerning personality throughout the lifetime [2–7]. While an earlier study showed that age largely influences personality [8], a later study showed that age per se does not cause personality instability [9]. Apart from deteriorations concerning family and social life it was found that work life, economic status and compromised health may lead to personality changes [10, 11]. Moreover, it has been shown that personality traits are associated with certain brain structures [12–14]. Accordingly, previous studies were able to show that personality changes are connected to neurodegenerative diseases (e. g. [15, 16]), such as Alzheimer’s disease [17–19] or mild cognitive impairment due to commencing dementia [19]. Furthermore, Alzheimer’s disease can be seen as a multi-causal concept of etiology encompassing biological, environmental, and biographical factors as well as the general state of health and personality traits [20]. For dementia patients, the experience of cognitive decline often results in negative emotions [21] and this affects the patient’s quality of life [22] as well as their caregivers’ [23]. Moreover, society as a whole suffers from the impact of dementia due to high societal costs [24]. Generally, dementia is still not remediable, but an early diagnosis may have several advantages [25] as early interventions might slow down the degenerative process [26–28]. Hence, identifying dementia as early as possible is beneficial and necessarily includes a better knowledge about pre-dementia stages. Due to a relation between personality and cognitive performance [29], different cognitive functions and their connection to personality traits were investigated.

### Prodromal stages of dementia

Dementia usually develops gradually progressing from subjective cognitive decline (SCD) to mild cognitive impairment (MCI) to dementia [30], while especially amnestic MCI (aMCI) progress to AD dementia with high probability [31–34]. While patients with SCD only subjectively notice declining cognitive abilities [30, 35], MCI describes an objectively measurable cognitive decline depending on age and educational level [31]. In case of memory deficits, it is labeled as amnestic MCI (aMCI), while non-amnestic MCI (naMCI) does not impair the patient’s memory [34, 36]. Even if the transition between normal aging and MCI is quite subtle [36], MCI can be seen as an abnormal state of cognitive detraction [36, 37] and as a precursor of AD [36, 38, 39]. In contrast to dementia [31, 40], MCI does not impair the individual’s activities of everyday life [31].

### Cognitive impairment and personality

Concerning personality, personality traits of the five-factor personality model were taken into account. This model encompasses five personality traits, namely neuroticism, extraversion, openness (to experience), agreeableness, and conscientiousness [1], which can be seen as the basic dimensions of personality including certain traits or facets that define these dimensions [11]. Regarding the connection between personality traits and the possible risk for generating MCI or AD later, previous studies partly show similar, but not consistent results [24, 41–43]. While some investigations showed that higher scores in neuroticism are a predictor for later AD [24, 41], another study stated that high neuroticism scores alone do not predict dementia, but the presence of both, high neuroticism and low extraversion [42]. Additional predictors for later generation of AD are lower scores in conscientiousness [24, 41, 43] and in openness [24]. High conscientiousness [43] and low neuroticism [44] are related to a reduction of AD risk and a decreased incidence of MCI. Furthermore, personality changes can be seen as predictors of future dementia [45]. Compared to the patients’ premorbid personality, AD patients are described by their caregivers as being more neurotic, less conscientious, less extraverted, and less open [17, 18] as well as less agreeable [17]. Such personality changes in patients with AD often appear at an earlier stage than the clinical diagnosis [25, 46]. By comparing MCI patients’ retrospectively examined premorbid personality to the current personality, significant changes in personality due to increasing neuroticism as well as decreasing extraversion and conscientiousness could be shown [47]. Considering personality as trait, extraversion scores are lower for AD patients [48] as well as for patients with SCD or MCI [49] than for healthy controls. Furthermore, AD patients report lower scores for openness and conscientiousness and higher neuroticism scores than healthy controls [48].

The groups’ mean ages of patients with SCD, naMCI, aMCI, and AD significantly differ from each other and increase in this order [50]. Concerning education, lower education goes along with a higher risk of dementia [51, 52], while there are largely no differences between the groups SCD, aMCI, naMCI, and AD and education [50]. Regarding education and personality traits of healthy individuals, openness to experience is positively, conscientiousness negatively correlated to education [53]. Results concerning intelligence and personality are quite heterogeneous. Within a previous investigation, no correlations between general intelligence and personality traits could be found [54]. In contrast to this, within further studies extraversion [55, 56], neuroticism, and conscientiousness [55] correlated negatively, openness positively [56] with intelligence. Concerning depressive symptoms, depression levels are significantly higher in patients with aMCI, naMCI [57, 58], and AD [57] than in healthy controls. Almost half of the patient group (SCD, aMCI, and naMCI) have depressive symptoms, in contrast to only about 17% of the healthy controls [59]. Regarding depression and personality, depressive symptoms were positively predicted by neuroticism [60, 61] and openness and negatively by extraversion [60].

Regarding these results of previous studies, we expected that there will be differences between investigated clinical groups concerning personality traits. Specifically, higher neuroticism scores and lower scores for extraversion, openness and conscientiousness were expected for patients with AD and those with aMCI than for individuals with SCD due to the similar symptom patterns of aMCIs and ADs [33]. Furthermore, the fact that patients with SCD and those with naMCI do not show memory complaints, while individuals with aMCI and those with AD actually do [34, 36], leaded to the assumption that differences between the two groups with memory problems and the two without will be smaller than between a group with memory complaints and a group without: hence, differences regarding personality traits were expected between patients with memory impairments (aMCI or AD) and those without memory complaints (SCD or naMCI). Due to a progressive development from SCD, to MCI and to AD [30] and the result that aMCIs are older than naMCIs [50], a positive correlation between age and investigation groups was expected. Moreover, age per se should be unrelated to personality [9].

## Methods

### Subjects and classification of the sample groups

The total sample of the investigation consisted of 133 participants, who came to the Department of Neurology, Medical University of Vienna due to memory complaints. Detailed sample sizes, demographical and clinical characteristics are shown in Table 1. From each participant written informed patient consent was obtained. The study protocol was approved by the Ethical Committee of the Medical University of Vienna and conducted in accordance with the Helsinki Declaration. All patients received a complete neurological examination, standard laboratory blood tests and psychometric testing. In most cases, a magnetic resonance imaging (MRI) scan of the brain was obtained. In determining significant cerebrovascular disease, both neuroimaging and clinical patient features were used. Inclusion and exclusion criteria were similar to other studies. Patients were excluded from the study if any of the following conditions applied: (a) evidence of stroke as determined by neuroradiologic and clinical examination, (b) history of severe head injury, (c) current psychiatric diagnosis according to ICD-10, however, patients with depressive symptoms were included because depressive symptoms often occur in elderly patients, (d) any medical condition that leads to severe cognitive deterioration including renal, respiratory, cardiac and hepatic disease, (e) age younger than 50 years, (f) patients with an Mini Mental State Examination (MMSE) [62] score <24. Neuropsychological testing was performed via the Neuropsychological Test Battery Vienna (NTBV), which mainly aims to diagnose elderly dementia [63]. Participants were assigned to one of the three groups SCD, naMCI and aMCIas follows: SCD is characterized by (a) a subjectively perceived degradation of memory abilities, (b) not objectively measurable cognitive deficits, and (c) medical help seeking of the affected person [30]. To diagnose MCI, Petersen criteria [33, 36] were applied. Therefore, measures of the individual’s cognitive functions via neuropsychological tests and clinical features were conducted [36]. The following criteria lead to an aMCI diagnosis: (a) the presence of memory complaint, which can be confirmed by an informant, (b) objective memory impairment, related to the individual’s age, (c) general cognitive functions are essentially preserved, (d) functional activities are intact, and (e) absence of dementia [36]. In case of all of these criteria being fulfilled, the individual got the diagnosis of aMCI, while in case of no memory decline, naMCI was diagnosed [36].

**Table 1:** Demographical and clinical characteristics of the investigated groups and the total sample

	Total	M (%)	F (%)	Age mean (SD)	Education mean (SD)	IQ mean (SD)	BDI-II mean (SD)	MMSE mean (SD)
SCD	31	58.1	41.9	67.21 (9.57)	12.98 (4.16)	113.48 (11.49)	9.87 (8.58)	28.74 (1.29)
naMCI	67	28.4	71.6	68.90 (9.41)	12.47 (4.09)	111.52 (12.59)	9.97 (8.05)	28.06 (1.60)
aMCI	35	60.0	40.0	66.66 (8.98)	13.94 (4.71)	112.00 (14.64)	9.79 (9.20)	27.69 (1.43)
Total Group	133	43.6	56.4	68.01 (9.32)	12.98 (4.29)	112.11 (12.83)	9.90 (8.41)	28.12 (1.53)

### Materials and measuring instruments

After the MMSE-screening [62], participants’ cognitive functions were investigated via the NTBV [63], which includes different cognitive areas: attention, executive functioning, language, and memory [57, 59, 63, 64]. According to [64], attention is measured by the Alters-Konzentrations-Test (AKT) [65], the Digit-Symbol Test—a subtest of the German Wechsler Adult Intelligence Scale-Revised (WAIS-R)—[66], the Symbol Counting task from the cerebral Insufficiency Test (c. I.) [67], and the Trail Making Test B (TMTB) [68]. By means of the Trail Making Test A (TMTA) [68], the Five-Point Test [69], the Maze Test from the Nürnberger Alters Inventar Test Battery (NAI) [70], the Stroop Test from the NAI [70], and the Interference Test from the c. I. [67], executive functions are assessed. Language functions are tested via the Semantic Verbal Fluency Test (SWT) [71], the Phonetic Verbal Fluency Test (PWT) [71], and the modified Boston Naming Test (BNT) [72]. Memory is tested via the Verbal Selective Reminding Test (VSRT) [73]. This test includes the four subtests Immediate Recall, Total Recall, Delayed Recall, and Recognition [73]. Although, all of the different domains have a corresponding z‑score and additionally a total z‑score across all tests [59, 63], raw data was used for further calculations due to the fact that the investigations of personality traits and the NTBV subtests are quite explorative. To measure personality the Big Five Plus One Persönlichkeitsinventar (B5PO) [74] was used. This includes the five personality traits neuroticism (called ‘emotional control’ within the B5PO), extraversion, openness, agreeableness, and conscientiousness of the current five-factor model/Big five personality model [75] and the additional personality trait empathy [74]. Due to the fact that within the B5PO the Rasch model is valid, internal consistency reliability is given [74]. Furthermore, theoretically based on the Big five personality model, the B5PO is also valid for the five personality traits of this model [56, 75]. Although percentile ranks of the B5PO are available, raw data—independently of gender, education, or age—were used for further calculations. To measure the individual’s intelligence, the Wortschatztest (WST) [76] was applied. The WST enables to investigate the verbal level of intelligence and the evaluation of the individual’s speech comprehension and allows as standardized vocabulary test an estimation of premorbid IQ [76]. Cronbach Alpha of 0.94 shows a high intern consistency reliability for the WST [76]. Concerning the validity correlations could show that the WST does hardly depend on the participant’s age (r = 0.08) and increases with higher educational levels (r = 0.60) [76]. The German version of the Beck depression inventory (BDI-II) [77] was used to measure the factor depression. The questions of the BDI-II concern the individual’s feelings during the last two weeks, and a BDI-II score >13 is an indication for clinically relevant depressive symptoms [77, 78]. Hence, seven people with SCD, eight persons with naMCI and 16 individuals with aMCI show clinically relevant depressive symptoms. Age and education were indicated by the total number of years.

### Analysis

Data were analyzed via the statistical program SPSS (version 24). The α‑level was set to *p* < 0.05. All data were investigated for normal distribution and equality of (co-)variance. Between-group differences concerning the personality traits were examined using multivariate analysis of variance (MANOVA) with the different personality traits as dependent variables and post-hoc tests, in spite of not normal distributed personality variables [79]. Potential confounding variables age, gender, education, IQ, and depression were examined with a multiple analysis of covariance (MANCOVA). Relations between diagnosis (SDC, naMCI, aMCI) and age, education, IQ, and depression were calculated by Spearman’s rank correlations, while group-wise correlations for these variables were calculated by Pearson product-moment correlations. Due to significant Kolmogorov-Smirnov tests for education, depression, and all of the personality traits, except of extraversion, Spearman’s rank correlations were used to examine relations between these variables, while relations between age, IQ, and extraversion were calculated by Pearson product-moment correlations. Spearman’s rank correlations were performed for age, education, IQ, depression, and the six personality traits over all groups as well as for group-wise correlations concerning naMCI and the variables conscientiousness, openness, and empathy. For the remaining correlations regarding naMCI and the group-wise correlations for SCD and aMCI, Pearson product-moment correlations were calculated due to normal distributions. Only correlation coefficients higher than 0.3 will be discussed due to at least moderate effects [80]. Relations between cognitive functioning and the different personality traits were investigated with Pearson product-moment correlations and Spearman’s rank correlations between the single tests of the Neuropsychological Test Battery Vienna (NTBV) and the particular personality traits. Due to the fact that only the personality trait extraversion is normally distributed, depending on the Kolmogorov-Smirnov tests of the NTBV subtests, some correlations between extraversion and certain NTBV subtests were calculated by Pearson product-moment correlations. Within the result report, Pearson product-moment correlations will be labeled, while the unlabeled results are based on Spearman’s rank correlations.

## Results

### Between-group differences concerning the personality traits (MANOVA)

The MANOVA reveals that over all investigation groups, four of the six examined personality traits significantly differ concerning the specific diagnosis. While agreeableness and emotional control/neuroticism do not show overall differences between the groups, scores for extraversion, conscientiousness, openness, and empathy vary significantly between the investigation groups (See Table 2 for details).

**Table 2:** MANOVA: Differences between the investigated groups and the personality traits

	*df*	*F*	*p*	*η*_*p*_^*2*^
Extraversion*	2	3.490	0.033	0.051
Agreeableness	2	0.037	0.964	0.001
Conscientiousness*	2	3.685	0.028	0.054
Emotional control	2	0.653	0.522	0.010
Openness**	2	5.813	0.004	0.082
Empathy**	2	8.253	<0.001	0.113

*df* degrees of freedom

*significant by F test at 5% of probability

**significant by F test at 1% of probability

**Fig. 1:**
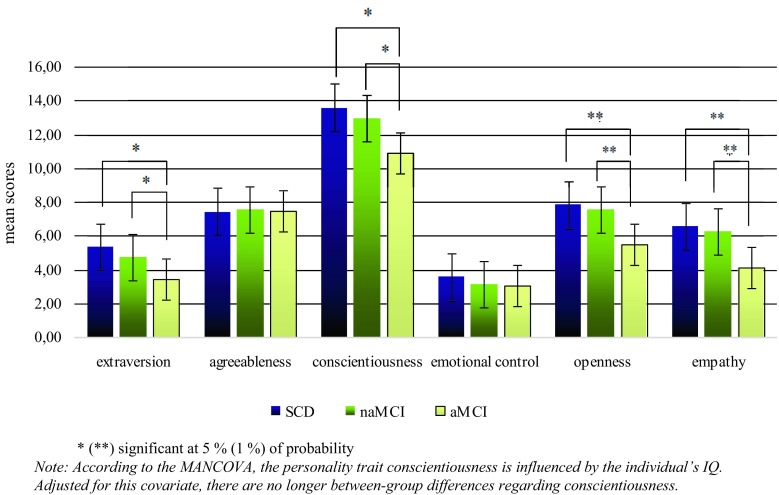
Post-hoc tests: Differences within the investigated groups and the personality traits (According to the MANCOVA, the personality trait conscientiousness is influenced by the individual’s IQ. Adjusted for this covariate, there are no longer between-group differences regarding conscientiousness; * (**) significant at 5% (1%) of probability)

Post hoc tests show that patients with aMCI have lower extraversion scores (*M* = 3.40; *SD* = 2.89) than those with naMCI (*M* = 4.76; *SD* = 3.33; *d*_Cohen_ = 0.427; *p* = 0.040) as well as patients with SCD (*M* = 5.35; *SD* = 3.04;* d*_Cohen_ = 0.659; *p* = 0.013), while there are no differences between SCD and naMCI. Similar patterns are also revealed by the scores for conscientiousness, openness, and empathy due to the fact that none of these personality traits show differences between SCD and naMCI. For conscientiousness, patients with aMCI have lower scores (*M* = 10.91; *SD* = 5.64) than those with naMCI (*M* = 12.93; *SD* = 3.87; *d*_Cohen_ = 0.444; *p* = 0.026) and those with SCD (*M* = 13.58; *SD* = 3.32;* d*_Cohen_ = 0.568; *p* = 0.013). SCD (*M* = 7.81; *SD* = 2.43; *d*_Cohen_ = 0.739; *p* = 0.004) as well as naMCI (*M* = 7.57; *SD* = 3.26; *d*_Cohen_ = 0.738; *p* = 0.002) scores for openness are higher than openness scores for patients with aMCI (*M* = 5.51; *SD* = 3.61). Similarly, aMCIs show lower empathy scores (*M* = 4.09; *SD* = 3.15) than patients with naMCI (*M* = 6.25; *SD* = 2.93;* d*_Cohen_ = 0.718; *p* < 0.001) and those with SCD (*M* = 6.55; *SD* = 2.23; *d*_Cohen_ = 0.892; *p* = 0.001). To sum up, as Fig. 1 shows, there are no differences between the investigated groups and the personality traits agreeableness and emotional control. Differences for extraversion, conscientiousness, openness, and empathy can only be seen for aMCI and SCD as well as for aMCI and naMCI. SCD and naMCI do not differ in any investigated personality traits.

### MANCOVA

Controlling for age, gender, education, IQ, and depression via MANCOVA, age (*η*_*p*_^*2*^ = 0.200; *p* = 0.000), gender (*η*_*p*_^*2*^ = 0.126; *p* = 0.013), IQ (*η*_*p*_^*2*^ = 0.101; *p* = 0.045), and depression (*η*_*p*_^*2*^ = 0.148; *p* = 0.003) influence the connection between personality and diagnosis. Adjusted by these factors, results and significances of the MANOVA largely do not change considerably for three of the four significant personality traits: extraversion (*F* = 3.524; *η*_*p*_^*2*^ = 0.054; *p* = 0.032), openness (*F* = 4.384; *η*_*p*_^*2*^ = 0.067; *p* = 0.014), empathy (*F* = 5.802; *η*_*p*_^*2*^ = 0.086; *p* = 0.004). Conscientiousness (*F* = 2.696; *η*_*p*_^*2*^ = 0.042; *p* = 0.071) does no longer reach significance. Separate MANCOVAs with only age, gender, education, IQ, or depression as covariates show that difference between the investigated groups and personality traits within the MANCOVA compared to the MANOVA do only change from significant to non-significant results for IQ and the personality trait conscientiousness (*F* = 2.948; *η*_*p*_^*2*^ = 0.044; *p* = 0.056).

### Correlations between personality and additional socioeconomic and psychological factors

Spearman’s rank correlations did not reveal any relations between diagnosis (SCD, naMCI, aMCI) and any of the investigated factors, namely age, education, IQ, or depression. Across all groups, education and IQ are positively correlated (*r* = 0.63; *p* < 0.001), education and depression negatively (*r* = −0.32; *p* < 0.001) A group-wise correlation between age, education, IQ, and depression shows a negative correlation between age and education for naMCI (*r* = −0.34; *p* = 0.005). Depression and education only correlate negatively for SCD (*r* = −0.42; *p* = 0.019). While there is a negative correlation between depression and IQ only for SCD (*r* = −0.40; *p* = 0.027), all of the investigated groups show a positive correlation between education and IQ (SCD: *r* = 0.71; *p* < 0.001; naMCI: *r* = 0.50; *p* < 0.001; aMCI: *r* = 0.64; *p* < 0.001). Furthermore, there is a positive correlation between age and IQ for aMCI (*r* = 0.38; *p* = 0.027).

Concerning the personality traits, none of the socioeconomic or psychological factors show at least moderate effects. Again, group-wise correlations show that only for naMCI a negative correlation between extraversion and depression (*r* = −0.38; *p* = 0.001) as well as between openness and depression (*r* = −0.30; *p* = 0.013) can be seen. Emotional control and education correlate positively for SCD (*r* = 0.37; *p* = 0.041) and aMCI (*r* = 0.43; *p* = 0.011). Additional group-specific correlations could be found between extraversion and age (*r* = 0.36; *p* = 0.047) and between agreeableness and depression (*r* = −0.37; *p* = 0.042) for SCD. Openness and age correlate positively for aMCI (*r* = 0.40; *p* = 0.019). Furthermore, emotional control and IQ (*r* = 0.48; *p* = 0.004), empathy and age (*r* = 0.35; *p* = 0.039), conscientiousness and age (*r* = 0.49; *p* = 0.003) as well as openness and IQ (*r* = 0.36; *p* = 0.040) are correlated for aMCI.

### Correlations between personality traits and cognitive functioning

Regarding the Neuropsychological Test Battery Vienna [63] and personality traits in Table 3, only small effects (<0.3 [80]). could be found.

**Table 3:** Correlations (in *r*) between cognitive functioning (NTBV) and personality traits

	Extraversion	Agreeableness	Conscientiousness	Emotional control	Openness	Empathy
AKT (time)	−0.07^a^	0.13	−0.06	0.05	−0.10	−0.03
AKT (mistakes)	0.27^**^	−0.06	−0.01	−0.25^**^	0.14	0.13
AKT (total/time)	0.00^a^	−0.12	0.06	−0.03	0.10	0.01
Digit-Symbol-Test	0.16^a^	−0.02	0.14	−0.01	0.21^*^	0.08
Symbols Counting (c. I.)	−0.06^a^	0.04	−0.04	0.01	−0.07	−0.01
TMTA	−0.03^a^	0.05	−0.06	−0.00	−0.07	−0.03
TMTB	−0.01	0.02	−0.09	−0.07	−0.03	−0.01
SWT	0.11^a^	−0.08	−0.02	−0.02	0.17^*^	0.01
PWT	0.08^a^	−0.01	0.07	−0.13	0.12	−0.02
BNT	0.12	−0.19^*^	−0.02	−0.12	0.05	0.00
VSRT Immediate Recall	0.14^a^	−0.04	0.03	−0.08	0.16	0.10
VSRT Total Recall	0.21^*,a^	−0.07	0.02	−0.11	0.24^**^	0.14
VSRT Delayed Recall	0.19^*^	−0.03	0.04	−0.13	0.23^**^	0.16
VSRT Recognition	0.15	0.03	0.14	0.01	0.19^*^	0.22^*^
Five Point Test	0.15^a^	−0.04	0.08	−0.01	0.24^**^	−0.00
Five Point Test—Perseverations	0.21^*^	−0.05	0.18^*^	−0.13	0.19^*^	0.18^*^
Stroop Test I (NAI-I) (time)	−0.09	0.10	−0.13	0.01	−0.08	−0.06
Stroop Test II (NAI-III) (time)	−0.06	−0.02	−0.06	−0.04	−0.11	−0.06
Stroop Test II (mistakes)	−0.05	0.04	−0.18^*^	−0.10	−0.09	−0.09
Stroop Test II (NAI-III)	0.07^a^	0.02	0.06	0.05	0.10	0.05
Labyrinth (time)	−0.09	0.03	0.05	−0.02	−0.09	0.05
Labyrinth (mistakes)	−0.05	−0.11	−0.03	−0.07	−0.16	−0.07
Labyrinth (total/time)	0.02^a^	−0.02	−0.04	0.03	0.11	−0.05
Interference (c. I.) (time)	0.01^a^	0.10	−0.04	−0.00	0.01	0.04
Interference (c. I.) (mistakes)	−0.16	−0.00	−0.00	−0.01	−0.08	−0.10
Interference (c. I.) (total/time)	−0.02^a^	−0.10	0.04	0.02	−0.01	−0.03

For AKT (time), AKT (mistakes), Symbols Counting (c. I.), TMTA, TMTB, Five Point Test—Perseverations, Stroop Test I (NAI-I) (time), Stroop Test II (NAI-III) (time) and Labyrinth (time), Interference (c. I.) (time) smaller values demonstrate a better performance

^a^calculated by Pearson product-moment correlations due to non-significant Kolmogorov-Smirnov tests (the unlabeled ones do not follow a normal distribution, hence Spearman’s rank correlations were performed)

*significant at 5% of probability

**significant at 1% of probability

## Discussion

Due to recent studies [2–7] which contradict the longstanding assumption that personality is quite stable over the lifetime [1], this investigation was meant to examine personality traits in dependence of changing health status—as one possible explication for personality changes [11]—in the course of time and age. While three of the six investigated personality traits—namely extraversion, openness, and empathy—showed significant differences between the groups SCD, naMCI, and aMCI, the remaining traits agreeableness, conscientiousness and emotional control did not reach significance after the MANCOVA. Regarding previous results, individuals with AD show increased neuroticism scores [17, 18, 25, 48]. Due to the fact that there is a progression from SCD to MCI to dementia [30], the expectation was that patients with aMCI—as the group that highly likely will progress to AD [31, 33, 34]—differ from those with SCD concerning neuroticism. This assumption could not be confirmed. Personality changes often appear at an earlier stage than the clinical AD diagnosis [25, 46], sometimes before symptoms can be noticed [46]. Hence, an explanation for the result that there are no differences between the cognitively impaired groups concerning neuroticism would be that this personality trait had already changed at the time when participants came to the Medical University of Vienna due to cognitive complaints. While previous investigations [48, 49] found decreased conscientiousness scores for AD patients, the expectation that there will be between-group differences concerning this personality trait could not be confirmed. In contrast to these studies [48, 49], our investigation also checked for covariates via MANCOVA and found out that IQ influenced the effects for conscientiousness. As expected, there were differences between the investigation groups regarding extraversion and openness. Individuals with aMCI consistently showed lower scores than those with naMCI and SCD. These results are in accordance with previous studies [48, 49], which found decreased extraversion and openness scores for patients with AD, keeping in mind that individuals with aMCI highly probably will convert AD [31, 33, 34]. The fact that there are no differences between SCD and naMCI represents the quite subtle transition between the groups [36]. Moreover, the symptoms for aMCI are comparable to those of AD [33], due to the fact that aMCI impairs the individual’s memory, while naMCI does not [34, 36].

Because of the fact that aMCI will highly probably progress to AD [31, 33, 34] and that aMCI and AD symptoms are pretty similar [33], it was expected that potential differences concerning personality traits between AD and aMCI as well as between naMCI and SCD would be smaller than between naMCI and AD, naMCI and aMCI, SCD and AD, and SCD and aMCI. Due to a lack of patients with AD that reached an MMSE-score >23, this group could not be investigated in the present study. Nonetheless, the results show that the only differences could be seen between either naMCI or SCD and aMCI. Therefore, it could be shown that the groups without memory impairment follow similar patterns regarding personality traits, while the group with memory disorders differs from the two other groups. Previous studies report a decrease for neuroticism, extraversion, openness, and conscientiousness for individuals with AD [48]. With the exception of the personality traits neuroticism and conscientiousness—which has already been discussed above—individuals with aMCI also had lower scores for extraversion and openness than patients with naMCI and SCD in the present study. For further investigations it would be interesting to include also healthy controls and—as far as possible—individuals with AD to examine, if and how patients with aMCI differ from those with AD concerning personality traits and if there is a gradual progress from healthy controls to cognitively impaired groups without memory disorder (SCD and naMCI) to those with memory impairment (aMCI and AD).

Due to the progressive development from SCD to MCI to AD [30] and considering the result that patients with aMCI are older than those with naMCI [50], a gradual increase in age from SCD to naMCI to aMCI was expected to be seen in the present investigation, too. In contrast to this assumption, no significant correlation concerning the three examined groups and age could be found. An explanation for these different results could be that in this investigation only individuals with an MMSE-score >23 were included. Especially patients with MCI probably do not always reach this cut-off. This might explain that there were no correlations between age and the three investigated groups.

The result that there is no correlation between age and the groups and the fact that the MANCOVA still shows significant differences between the investigated groups regarding extraversion, conscientiousness, openness, and empathy after adjusting for age demonstrate that age per se is not related to personality. This goes along with previous findings, which also show that age as such does not influence personality [9].

Generally, the correlations between the cognitively impaired groups and age, education, IQ, and depression as well as between the different personality traits and the socioeconomic and psychological variables revealed quite heterogeneous results. Regarding previous investigations, this is not surprising. The assumption that there would be a correlation between depression and the cognitively impaired groups, mediated by neuroticism, could not be confirmed. Specifically, higher depression scores were expected for individuals with aMCI than for those with SCD, if neuroticism had also been higher for aMCI than for SCD due to a positive correlation between neuroticism and depression in previous studies [60, 61]. But as the current investigation did not reveal differences between the cognitively impaired groups and neuroticism, there was also no correlation between the variables group and depression. As neuroticism could not mediate, the fact that there were no correlations between the investigated groups and depression goes along with previous results [57].

It is necessary to take into account that the investigation took place in Vienna and hence, included Central Europeans. As culture actually affects the expression of personality [1], the investigation can give evidence for personality traits of cognitively impaired groups in Central Europe and further research within other cultures would be interesting. Moreover, the investigated patients already received medical aid or seeked for medical help. This might lead to a special subgroup of the population, which does not include patients, who do not notice cognitive declines or who do not want to get any medical help, although they do actually notice changes. It would be imaginable that these groups (medical help seeking/not medical help seeking patients) also differ regarding personality traits. But due to the fact that the participation in this investigation is voluntary, it would be difficult to examine also patients, who do not look for medical aid despite cognitive decline. Another problem of the present study is that AD could not be investigated due to the exclusion criteria that the MMSE score had to be >23. But it has to be mentioned that a complete assessment encompassing the NTBV [63], the B5PO [74], the WST [76], and the BDI-II [77] would have been too exhausting for most of the individuals with an MMSE score <24. Moreover, a limitation of correlation studies is that results do not show causality. Hence, it is unclear, how personality and cognitive impairment influence each other and further investigations about personality and cognitive impairment would be interesting to clarify how they are connected due to the possibilities that personality could cause cognitive impairment or the other way round.

Knowledge about personality and prodromal stages of dementia might be helpful to identify dementia as early as possible. In the present study we found that patients with SCD did not differ from those with naMCI concerning different personality traits, patients with aMCI showed significantly lower scores for extraversion, openness, and empathy than patients with SCD as well as patients with naMCI. Thus, cognitively impaired groups mainly differ concerning personality traits depending on whether they do show memory decline or not.
